# HOW OLD DO YOU FEEL? CONSIDERING THE CONTEXTS, DYNAMICS, AND ASSESSMENT OF SUBJECTIVE AGE

**DOI:** 10.1093/geroni/igac059.641

**Published:** 2022-12-20

**Authors:** Anna Kornadt, Jennifer Bellingtier

## Abstract

How old people feel is a highly effective predictor of later life health and well-being. Despite a wealth of research, the developmental dynamics of the construct as well as its antecedents and consequences are not well understood. Our symposium brings together research that models dynamic trajectories in subjective age over long- and short periods of time and links it to psychological constructs and objective indicators of health and functioning. First, Weiss and colleagues present longitudinal findings of subjective age trajectories in a lifespan sample that highlight the reciprocal dynamics between subjective age and social contexts. Bellingtier and colleagues link the age people feel on a daily basis to the age people want to feel and find that when people felt closer to the age they desired, their affect was more positive. Rupprecht and colleagues measured subjective age as well as affect, stress and physical activity on 21 consecutive days. Data attest to the relevance of daily experiences for subjective age. In a similar approach, Tingvold and colleagues show the relationship of momentary subjective age with subjective and physiological stress in late-midlife adults’ daily lifes. Finally, Touron and Hughes found that momentary fluctuations in subjective age are associated with current task engagement and enjoyment. Together, the findings show that innovative perspectives and research designs are needed to understand how people respond to the question “How old do you feel” and why it predicts how well they actually age.

